# Clinical characteristics of breast cancer patients admitted to academic surgical wards in Tehran, Iran: an analytical cross-sectional study

**DOI:** 10.1186/s12905-023-02637-0

**Published:** 2023-09-25

**Authors:** Reza Pourriahi, Ramesh Omranipour, Sadaf Alipour, Leila Hajimaghsoudi, Negar Mashoori, Adel Yazadnkhah Kenary, Mandana Motamedi, Mahsa Tavakol, Mahta Mohammadzadeh, Shiller Hessamiazar, Samira Shabani, Fatemeh Mahmoodi, Mohammadreza Mirzaee Goodarzi, Bita Eslami

**Affiliations:** 1 Breast Diseases Research Center, Cancer Institute, Tehran University of Medical Sciences, Imam Khomeini Complex Hospital, 2nd Floor, Sadaf Building, Keshavraz Blvd, Tehran, 1419733141 Iran; 2 Department of Surgical Oncology, Tehran University of Medical Sciences, Tehran, Iran; 3 Department of Surgery, Arash Women’s Hospital, Tehran University of Medical Sciences, Tehran, Iran; 4 Department of Surgery, Shahid Madani Hospital, Alborz University of Medical Sciences, Karaj, Iran; 5 Sina Trauma and Surgery Research Center, Tehran University of Medical Sciences, Tehran, Iran; 6 Breast Cancer Research Center, Motamed Cancer Institute, ACECR, Tehran, Iran; 7 Department of Neurology, Tehranpars Hospital, Tehran, Iran; 8 Fatemehzahra Hospital, Iran University of Medical Sciences, Tehran, Iran; 9 Medical School, Tehran University of Medical Sciences, Tehran, Iran

**Keywords:** Breast Cancer, Cross-Sectional study, Multicenter study, Prevalence, Iran

## Abstract

**Background:**

Breast cancer (BC) is the most commonly diagnosed cancer and the leading cause of cancer death among women. Knowledge of the clinical characteristics of BC in a population may be informative for disease prediction or diagnosis and for developing screening and diagnostic guidelines. This study aimed to evaluate the clinical characteristics of female patients with BC who were admitted to academic surgical wards in Tehran, Iran.

**Methods:**

In this cross-sectional study, demographic information and clinical characteristics of Iranian females with BC who had undergone breast surgery from 2017–2021 in four academic Breast Surgery Units were extracted from medical files and recorded via a pre-designed checklist.

**Results:**

A total of 1476 patients with a mean age of 48.03 (± 11.46) years were enrolled. Among them, 10.4% were aged less than 35. In younger patients, Triple-negative and Her2-enriched subtypes of BC were significantly higher compared to older ones. Overall, 85.7% of tumors were invasive ductal carcinoma, 43.3% were grade 2, 41.4% were located in the UOQ, and 65.2% had presented with mass palpation. The mean pathologic tumor size was 28.94 mm, and the most common subtype was luminal B.

**Conclusions:**

Many characteristics of breast cancer in this study were similar to other countries and previous studies in Iran. However, a higher proportion of young BC compared with Western countries, and even with older studies in Iran, suggest a trend toward lower age for BC in recent years. These results indicate the need for preventive measures and screening in Iranian women at a younger age.

## Background

According to the World Health Organization (WHO) in 2019, cancer is the first or second leading cause of death before the age of 70 in 112 of 183 countries and ranks third or fourth in a further 23 countries [1]. Based on the estimates of the GLOBOCAN 2020, breast cancer (BC) is the most commonly diagnosed cancer and leading cause of cancer death among women and its incidence in developed communities is higher than in developing ones. This may reflect the higher frequency of risk factors related to breast cancer such as low parity, higher age at first pregnancy, use of oral contraceptive pills (OCP), use of hormone replacement therapy (HRT), high-calorie diet, and alcohol consumption in developed countries [2, 3].

In comparison to Europe and the United States, the incidence and prevalence of BC are lower in Asia. However, the mortality rate of BC in low-income countries is higher than in high-income ones [4–6]. Based on previous studies, BC occurs sooner in Asia (around 40–50 years old) than in Western countries (about 60–70 years old) [7]. In addition, the proportion of young patients aged less than 35 years is about 10% in developed nations, vs. 25% in developing areas [8]. In Iran, BC accounts for about 32 percent of all women diagnosed with cancer, and it is defined as the sixth major cause of death [3]. According to the Iranian National Cancer Registry (INCR), the age-standardized incidence rate (ASIR) of primary BC is 27.4 per 100,000 persons per year in Iran [9].

The nature, incidence, and prognosis of BC vary in accordance with the patient’s characteristics, such as the patient’s age, menopausal status, and family history; as well as the tumor characteristics [10]. Recent studies conducted in Iran demonstrate that family history of BC, low parity, employment, and oral use of contraceptives are associated with an elevated risk of BC [5, 9].

Knowledge of the distribution and frequency of clinical characteristics of BC in a population may be informative for disease prediction or diagnosis, and assists in decision-making for treatment purposes, or developing screening and diagnostic guidelines [10]. Furthermore, BC would be a more serious health issue and may put a strain on the healthcare systems of middle-income countries like Iran in the near future [5, 9].

This study aimed to evaluate the clinical characteristics of female patients with BC in Iran. As Tehran University of Medical Sciences is the leading academic and referral center in the country, we gathered data of all patients who had undergone breast cancer surgery during a recent 4-year period in all Breast Surgical Units of the University to report the surgical approach and evaluate their main clinical and histological features; and explore the proportion and characteristics of young BC.

## Methods

### Study design

This multicenter cross-sectional study was approved by the Ethics Committee of Tehran University of Medical Sciences (IR.TUMS.IKHC.REC.1400.006) and was conducted on women diagnosed with BC who had undergone breast surgery from 2017 to 2021. Informed consent had been obtained from all breast cancer patients before surgery in order to use the information for research projects. The project was run according to the ethical principles of the Declaration of Helsinki. All active Breast Surgery Units of the University, which include four academic hospitals (Imam Khomeini Hospital, Cancer Institute, Arash Women's Hospital, and Sina Hospital) affiliated with Tehran University of Medical Sciences, Tehran, Iran, have participated in the present study.

### Participants' characteristics

In this study, data of 1476 females with BC who had undergone breast surgery were extracted. The sources of this information were medical files, pathologic results as well as the electronic archive system available in some hospitals. A similar pre-designed checklist was used in all centers. The checklist contained three parts. The first part variables included demographic and reproductive characteristics, the second comprised breast cancer information, and the last part covered breast surgery data.

Age, weight, height, smoking habit and alcohol consumption, age at menarche, age at first pregnancy, parity, menopausal status, age and cause of menopause, OCP use, history of abortion and family history of breast and ovarian cancer were extracted. Clinical characteristics of the disease consisted of presenting symptoms, tumor size, axillary lymph node status, core needle biopsy results, metastasis status and sites, findings of breast and axillary lymph node surgeries, tumor grade, and treatment types.

Tumors had been examined histologically by board-certified pathologists dedicated to breast diseases in the four academic centers. The histologic type had been assigned according to the 5th edition of the WHO Classification of Tumors of the Breast, in 2019 [11]; tumor grade had been classified as G1, G2, or G3 according to the Modified Bloom-Richadson Grading System [12]. BC was categorized into four molecular subtypes, including Luminal A (estrogen receptor (ER) /progesterone receptor (PR) + , Her2 negative, Ki 67% ≤ 15%), Luminal B (ER + and/or PR + , HER2 + or HER2 − with Ki67 > 15%), triple-negative/basal-like (ER − , PR − , HER2 −), and HER2-enriched type (ER − , PR − , HER2 +) [13].

In this study, the tumor sites have been categorized as the upper outer quadrant (UOQ), the upper inner quadrant (UIQ), the lower outer quadrant (LOQ), the lower inner quadrant (LIQ), as well as multicentric tumors defined as tumors in different quadrants or more than 5 cm apart in one breast [14, 15]. BC surgeries have been categorized as breast-conserving surgery (BCS), mastectomy, and others, which include flap reconstructions, nipple reconstructions, and excisional biopsy.

In cases with incomplete information, the patient’s records were obtained and completed through a telephone interview. Patients with incomplete medical records that could not be retrieved by phone calls were excluded from the study.

### Statistical analysis

Statistical analyses were conducted using SPSS software version 22 for Windows (IBM Inc, NY). Continuous data are presented as mean ± standard deviation (SD) and categorical data as frequency counts (percentages). Patients were categorized into two age groups: patients less than 35 years old, and those above or equal to 35 years old; with the purpose of comparing the tumor and clinical characteristics of BC in these two age populations. The Chi-square test was used to determine the differences between the qualitative variables of the two groups. The normality of Ki-67% was tested using Kolmogorov–Smirnov Test. The results showed the Ki-67% level was not normally distributed (*p*-value < 0.001). Therefore, a comparison of Ki-67% between the two groups was conducted using Mann–Whitney U-test. *P* values < 0.05 were considered statistically significant.

## Results

The final analysis of 1476 female patients showed a mean age of 48.03 (± 11.46) years (range: 17 to 86 years). The baseline information of the patients is presented in Table 1, in detail. Our results showed that about half (50.9%) of the patients were postmenopausal, and about 31% of these women had experienced menopause naturally. About 50% of these women had a history of abortion and 39.8% had a previous history of OCP usage. Also, 24.1% of women had a family history of breast or ovarian cancer.

**Table 1:** Baseline Characteristics of Patients

**Baseline Characteristics (Quantitative Variables)**			
	**Minimum**	**Maximum**	**Mean ± SD**
**Age (yrs)**	17	86	48.03 ± 11.46
**BMI (kg/m**^**2**^**)**	13.71	52.34	27.20 ± 4.54
**Age at menarche (yrs)**	9	22	13.39 ± 1.54
**Age at first pregnancy (yrs)**	12	47	22.26 ± 5.45
**Parity (n)**	0	16	2.74 ± 2.11
**Breastfeeding duration (mo)**	0	264	44.80 ± 41.83
**Baseline Characteristics (Qualitative Variables)**		**Frequency**	**Valid Percent (%)**
**Menopausal status**	**Menopausal**	602	50.9
	**Premenopausal**	580	49.1
	**Total**	1182	100
**Causes of menopause**	**Natural menopause**	322	31.1
	**Surgical**	71	6.9
	**Medical**	83	8
	**Unknown reason**	558	54
	**Total**	1034	100
**Abortion**	**Yes**	191	49.7
	**No**	193	50.3
	**Total**	384	100
**OCP consumption**	**Yes**	422	39.8
	**No**	639	60.2
	**Total**	1061	100
**Smoking**^**a**^	**Yes**	105	11.8
	**No**	782	88.2
	**Total**	887	100
**Hormone replacement therapy**	**Yes**	32	3.3
	**No**	705	95.7
	**Total**	737	100
**Family history of BC or OC**	**Yes**	303	24.1
	**No**	953	75.9
	**Total**	1256	100

*BMI* Body mass index, *SD* Standard deviation, *BC* Breast Cancer, *OC* Ovarian cancer

^a^Active and passive smokers

Table 2 presents the clinical characteristic of breast cancer in the study sample. At the time of diagnosis, 968 patients (85.7%) had invasive ductal carcinoma (IDC). Regarding tumor locations, the most frequent site was the UOQ (474 cases, 41.4%) and 98 cases (8.5%) had multicentric tumors. The most frequent symptom was mass palpation (*n* = 962, 65.2%) followed by nipple retraction (70 cases, 4.7%). The mean pathologic tumor size was 28.94 (± 21.76), and the majority of patients (43.7%) were diagnosed when the pathologic size of the tumor was between 20–50 mm. The most frequent tumor grade was G2 (43.3%).

**Table 2:** Clinical characteristics of breast cancer disease in study population

**Variables**	**Categories**	**N (%)**
**Breast cancer side**	Right	719 (48.7)
	Left	745 (50.5)
	Bilateral	2 (0.1)
	Missing	10 (0.7)
**Pathologic tumor size**	Less than 20 mm	455 (36.3)
	20 – 50 mm	547 (43.7)
	More than 50 mm	251 (20)
**Symptoms**	Mass palpation	962 (65.2)
	Nipple retraction	70 (4.7)
	Dimpling	48 (3.3)
	Edema	39 (2.6)
	Erythema	35 (2.4)
**Tumor site**	UOQ	474 (41.4)
	UIQ	154 (13.4)
	LOQ	98 (8.5)
	LIQ	62 (5.4)
	Multicentric	98 (8.5)
**Grade**	G1	216(16.4)
	G2	571 (43.3)
	G3	300 (22.7)
**LVI**	Yes	582 (51)
	No	560 (49)
**Histologic type**	IDC	968 (85.7)
	DCIS	91 (8)
	ILC	49 (4.3)
	Others	21 (2)
**Molecular Subtype**	Luminal B	439 (46.3)
	Luminal A	279 (29.4)
	Triple-negative (TN)	132 (14)
	Her2-enriched	98 (10.3)
**Metastatic site**	Bone	64 (4.6)
	Liver	28 (2)
	Lung	27 (1.9)
	Skin	21 (1.5)
	Other	14 (1)
**Breast surgery type**	Breast-conserving surgery	833 (56.4)
	Mastectomy	629 (42.6)
	Mastectomy + reconstruction	71 (4.8)
**Axillary surgery type**	ALND	610 (46.7)
	SLNB	696 (53.3)
**Adjuvant treatment**	Chemotherapy	760 (51.5)
	Radiotherapy	451 (30.5)

Data are presented as number with percentages in parenthesis

*UOQ* Upper outer quadrant, *UIQ* Upper inner quadrant, *LOQ* Lower outer quadrant, *LIQ* Lower inner quadrant, *IDC* Invasive ductal carcinoma, *DCIS* Ductal carcinoma in situ, *ILC* Invasive lobular carcinoma, *ALND* Axillary lymph node dissection, *SLNB* Sentinel lymph node biopsy

Out of 948 tumors, in order of prevalence, 439 (46.3%) were Luminal B, 279 (29.4%) were Luminal A, 132 (14%) were triple-negative (TN) and 98 (10.3%) were Her2-enriched. Out of 1476 cases, 126 (8.7%) had at least one metastatic site. The most common sites of metastasis were bone (64 cases, 4.6%), liver (28 cases, 2%), lung (27 cases, 1.9%), skin (21 cases, 1.5%), and other sites (14 cases, 1%).

More than half of the 1476 recorded patients (*n* = 760, 51.5%) had received chemotherapy, and 451 (30.5%) had undergone radiotherapy. The most frequent surgery was BCS (*n* = 833, 56.4%) followed by mastectomy in 42.6% (*n* = 629) and the remaining had undergone mastectomy with reconstruction (*n* = 71, 4.8%). Axillary lymph node dissection (ALND) had been performed for 610 (46.7%) and sentinel lymph node biopsy (SLNB) for 696 subjects (53.3%).

Our results represented that 154 patients (10.4%) were aged less than 35, and there was a significant difference between the molecular subtype of BC considering patients’ age (*P* value: 0.009). In triple-negative and Her2-enriched BC, the proportion of patients aged less than 35 was higher than those 35 years of age or above (Table 3). Furthermore, Ki67% was significantly higher in younger BC patients. Pregnancy-associated BC (PABC) was diagnosed in 17 women (1.2%).

**Table 3:** Comparison of variables between patients based on age groups (< 35 vs ≥ 35 years)

**Variables**		**<35 years**	**≥35 years (*n=*1322)**	***P*-value**
		**(*n=*154)**		
**Family history of BC or OC**	Positive	28 (21.5%)	275 (24.4%)	0.47
	Negative	102 (78.5%)	851 (75.6%)	
**Grade**	G1	15 (15.3%)	201 (20.3%)	0.055
	G2	46 (46.9%)	525 (53.1%)	
	G3	37 (37.8%)	263 (26.6%)	
**LVI**	Yes	57 (53.8%)	525 (50.7%)	0.54
	No	49 (46.2%)	511 (49.3%)	
**Pathologic size category (mm)**	≤20	45 (36.6%)	410 (36.3%)	0.25
	21-50	47 (38.2%)	500 (44.2%)	
	>50	31 (25.2%)	220 (19.5%)	
**Histologic type **	IDC	94 (87.9%)	874 (85.4%)	0.54
	DCIS	9 (8.4%)	84 (8.2%)	
	Others	4 (3.7%)	66 (6.4%)	
**Molecular subtype**	Luminal A	14 (15.2%)	265 (28.8%)	**0.009**
	Luminal B	41 (44.6%)	398 (43.3%)	
	Luminal X	5 (5.4%)	59 (6.4%)	
	Her2-enriched	16 (17.4%)	82 (8.9%)	
	Triple negative	16 (17.4%)	116 (12.6%)	
**Ki-67 (%)**		30.86 ± 22.13	24.88 ± 19.28	0.01*

Luminal X means ER and/or PR positive, Her-2 negative, Unknown Ki-67%. *US* Ultrasonography, *BC* Breast Cancer, *OC* Ovarian Cancer, *LVI* Lymphovascular Invasion, *CNB* Core Needle Biopsy, *IDC* Intra-ductal carcinoma, *DCIS* Ductal Carcinoma Insitu, *ILC* Intra-Lobular Carcinoma, *LCIS* Lobular Carcinoma Insitu

*P*-value refers to Chi-square test in categorical variables. Mann–Whitney U-test was used for comparison of Ki-67% between two-groups

## Discussion

The review of medical records of 1476 breast cancer patients who had undergone breast surgery in four academic hospitals of the largest university (TUMS) in Iran, between 2017–2021 showed, the mean age of BC patients who undergone surgery is lower than 50 years old and about 10% of patients were aged less than 35. Palpation of the mass, between 20 and 50 mm, was the most common symptom in patients at the time of diagnosis. More than half of the patients had BCS surgery. The luminal subtype of BC was more prevalent in the total population. However, triple-negative and Her2-enriched BC were the most prevalent subtypes in younger patients. Since these hospitals are considered referral centers, it seems that the results of the current study have high generalizability.

BC is characterized by several clinical and histological types. Large differences have been reported in the age of onset, stage at presentation, clinical manifestations, and prognosis of BC between various countries, mainly between BC patients from the Middle East or North Africa, and those from the Western populations [16, 17]. According to the American Cancer Society, BC mainly occurs in women middle-aged and older, with a median age at the time of diagnosis of 62 years (available on: www.cancer.org/cancer/types/breast-cancer/about/how-common-is-breast-cancer.html). Our study result supported a previous report which showed that BC incidence peaks among women in their forties in Iran, whereas in the United States and Europe, it peaks among women in their sixties [17]. In the present study, the mean age of the patients was 48 years, however, the previous study by Taghipour et al. reported a mean age of 50 ± 12.9 years among 566 Iranian BC patients between 2008 and 2014 [18]. Comparing the average age in the present study and Taghipour's study raises the possibility that the average age of breast cancer in our country is decreasing, and this point shows the necessity of preventive strategies and breast cancer awareness in our country. Furthermore, another study on very young BC in Tehran, Iran from 1979 to 2012 by Alipour et al. [19] revealed that 1.17% of BC patients attending a referral center throughout 33 years were 25 years old or less; whereas in our study, 1.4% of patients were in these ages at the time of BC diagnosis. Thus, the proportion of BC patients aged less than 25 years was higher in the present study compared with the findings of Alipour et al.; this also is in favor of the decreasing age of BC in Iran.

Based on the present study results, about one-fourth of patients reported a positive history of OCP usage. In addition, more than one-third of recruited patients had a positive familial history of BC or OC. In a nested case–control study derived from the Golestan Cohort., long-term use of OCP and a positive familial history were reported in 28.3% and 34.3% of BC patients, respectively [16]; these findings are consistent with the present study.

In consistence with our results, many studies reported that the left breast is at greater risk of developing cancer and the UOQ is the most common location of breast cancer [20–22]. It might be important to pay attention to the area of primary involvement because some evidence showed that the survival of BC patients with UOQ involvement is significantly better than tumors located elsewhere in the breast [23–25].

Globally, IDC constitutes about 70–80 percent of all BCs [25]. The results of our study also show that IDC is the most common type of BC in our population. Similar to our study, a previous study in Iran reported that grade 2 BC is the most prevalent grade, and that grade 1 is the least commonly seen [18].

The most common subtype of BC in the present study was Luminal B, followed by Luminal A, TNBC, and Her2-enriched. The frequency of molecular subtypes in our study followed the same order as those of Mighri et al. [22], Al Tamimi et al. [26], and Caldarella et al. [27]. However, these results are inconsistent with some previous studies conducted in Algeria, Egypt, Japan, and the USA [28–31]. These controversies may be due to different cut-off values set for Ki-67 in the stratification of luminal BCs.

In our study, 8.7% percent of patients were diagnosed with metastatic BC. Bone metastasis was reported as the most common site, followed by metastasis to the skin, liver, lung, and brain. A recent study was performed by Anwar et al. on 1,329 females with BC to investigate risk factors, patterns, and distribution of bone metastases and skeletal-related events in high-risk BC patients [31]. Their findings showed a rate of about 18.5 percent for metastatic BC (higher than ours), but like our results, bones were the most common site of metastasis in their analysis. As our population consisted of women admitted to surgical wards, the rate of metastatic BC is not demonstrative for all BCs. Bone metastasis has the best prognosis among metastatic sites [32], and curative surgery is considered in many cases. The non-expected high rate of skin metastases is because these patients are occasionally referred for surgical excision of the skin lesions.

Worldwide, BCS is defined as the most common surgical procedure for early-stage BC [33]. An institutional review board in the United States reported that the mastectomy rate (including bilateral mastectomy and reconstruction) has increased slowly during 1994–2007 (33%–44%) [34]. A cohort study in the USA on 21,869 BC patients from 1998 to 2007 also showed an increased rate of mastectomy for early-stage BC treatment in all age groups [35]. In our study, BCS was more commonly performed (56.4% for BCS vs. 42.6% for mastectomy). The rate of mastectomy in our country is similar to the US report in 2007 (44%) [34] and is lower than Saudi Arabia (62.4%) [33]. In BC women of Saudi Arabia, the predictor of mastectomy was increased tumor size, stage, and HER-2 positivity [33]. This worldwide increasing trend of mastectomy emphasizes the urgent need for early detection screening protocol to move towards BCS. The geographical difference in the decision for BCS or mastectomy in early-stage BC has been observed in other studies as well. One study in Iran has indicated that Iranian general surgeons avoided BCS and their major reason (46.3%) was uncertainty about the result of conservative therapy [36]. It seems that the rate of mastectomy is still high; therefore, holding breast surgery fellowship courses and training classes for general surgeons and taking advantage of consultation in a multidisciplinary team (MDT) breast is highly recommended.

One study showed that the risk of death from breast cancer has increased sharply in women younger than 35 years. Therefore, they recommended that age less than 35 years is a reasonable cut-off for defining young age-onset breast cancer [37]. In the present study, we compared the clinical characteristics of BC patients younger or older than 35. Our results showed that tumor size was larger in younger patients and Ki-67% as a proliferative factor of breast cancer was significantly higher in those patients. Furthermore, the rate of good prognosis tumors (Hormone-receptor positive) was lower in patients younger than 35. These results are consistent with several previous studies [38–40].

The current study has the advantage that it was conducted in several academic and active referral hospitals, and its results seem to be generalizable, although, this study is ongoing on a wider scale with a large sample size. However, this study had some limitations. Despite the young age of patients, data about genetic testing were scarce and therefore were not included in this study. Also, we did not have information about the ethnicity of the patients, but this could provide interesting findings.

## Conclusions

This study highlights the epidemiological and clinical characteristics of BC in Iranian women, with a special focus on factors that were not well defined in previous records in the Iranian population. In line with previous studies, invasive ductal carcinoma was the most common histologic type, grades 2 and 3 constituted the highest proportion of tumors. Luminal B was the most frequent molecular subtype, followed by Luminal A. Young women constituted a higher proportion of cases compared with older studies, suggesting a trend toward lower age for BC in recent years. These results indicate the need for more attention and preventive measures and screening in Iranian women before 50 years.

## Data Availability

The datasets analysed during the current study are available from the corresponding author on reasonable request.

## Abbreviations

WHO: World Health Organization
BC: Breast cancer
OCP: Oral contraceptive pills
HRT: Hormone replacement therapy
NCR: National Cancer Registry
ASIR: Age-standardized incidence rate
ER: Estrogen receptor
PR: Progesterone receptor
UOQ: Upper outer quadrant
UIQ: Upper inner quadrant
LOQ: Lower outer quadrant
LIQ: Lower inner quadrant
BCS: Breast-conserving surgery
SD: Standard deviation
IDC: Invasive ductal carcinoma
ALND: Axillary lymph node dissection
SLNB: Sentinel lymph node biopsy
PABC: Pregnancy-associated BC
MDT: Multidisciplinary team

## References

[1] World Health Organization (WHO). Global Health Estimates 2020: Deaths by Cause, Age, Sex, by Country and by Region, 2000–2019. WHO; 2020. https://www.who.int/data/gho/data/themes/mortality-and-global-health-estimates/ghe-leading-causes-of-death. Accessed 11 Dec 2020.

[2] American Cancer Society (2022). Cancer Facts and Figures 2022.

[3] Sung H, Ferlay J, Siegel RL, Laversanne M, Soerjomataram I, Jemal A, Bray F (2021). Global cancer statistics 2020: GLOBOCAN estimates of incidence and mortality worldwide for 36 cancers in 185 countries. Cancer J Clin.

[4] Farhood B, Geraily G, Alizadeh A (2018). Incidence and mortality of various cancers in Iran and compare to other countries: a review article. Iran J Public Health.

[5] Sharifian A, Pourhoseingholi MA, Emadedin M, Nejad MR, Ashtari S, Hajizadeh N (2015). Burden of Breast Cancer in Iranian Women is increasing. Asian Pac J Cancer Prev.

[6] Medhin LB, Tekle LA, Fikadu DT, Sibhatu DB, Gebreyohans SF, Gebremichael KH, et al. Incidence of Breast Cancer in Eritrea: A Retrospective Study from 2011 to 2017. Int J Breast Cancer. 2019;1. 10.1155/2019/8536548. 10.1155/2019/8536548 PMC6633959 31355003

[7] Wong FY, Tham WY, Nei WL, Lim C, Miao H (2018). Age exerts a continuous effect in the outcomes of Asian breast cancer patients treated with breast-conserving therapy. Cancer Commun.

[8] Pathy NB, Yip CH, Taib NA, Hartman M, Saxena N, Iau P (2011). Breast cancer in a multi-ethnic Asian setting: results from the Singapore-Malaysia hospital-based breast cancer registry. Breast.

[9] Dolatkhah R, Somi MH, Jafarabadi MA, Hosseinalifam M, Sepahi S, Belalzadeh M, et al. Breast cancer survival and incidence: 10 years cancer registry data in the Northwest, Iran. Int J Breast Cancer. 2020;2020. 10.1155/2020/1963814 PMC7211235 32411480

[10] Phipps AI, Li CI (2010). Breast cancer biology and clinical characteristics. In Breast cancer epidemiology.

[11] Tan PH, Ellis I, Allison K, Brogi E, Fox SB, Lakhani S, et al. WHO Classification of Tumours Editorial Board. The 2019 World Health Organization classification of tumours of the breast. Histopathology. 2020;77(2):181-5. 10.1111/his.14091 32056259

[12] Yap KK, Efiom-Ekaha DN (2011). Is modified Bloom-Richardson grade a reliable predictor of breast cancer recurrence?. J Clin Oncol..

[13] Winchester DJ, Winchester DP. Breast Cancer. Pmph Bc Decker. (2006) ISBN:1550092723.

[14] Siotos C, McColl M, Psoter K, Gilmore RC, Sebai ME, Broderick KP (2018). Tumor Site and Breast Cancer Prognosis. Clin Breast Cancer.

[15] Yanagawa M, Ikemot K, Kawauchi S, Furuya T, Yamamoto S, Oka M (2012). Luminal A and luminal B (HER2 negative) subtypes of breast cancer consist of a mixture of tumors with different genotype. BMC Res Notes.

[16] Alipour S, Omranipour R, Malekzadeh R, Poustchi H, Pourshams A, Khoshnia M (2019). A Case-control study of breast cancer in Northeast of Iran: The Golestan Cohort Study. Arch Iran Med.

[17] GLOBOCAN 2020: Global Cancer Observatory. International Agency for Research on Cancer 2022. https://gco.iarc.fr/today/online-analysispie?v=2020&mode=cancer&mode_population=continents&population=900&populations=900&key=total&sex=0&cancer=39&type=0&statistic=5&prevalence=0&population_group=0&ages_group%5B%5D=0&ages_group%5B%5D=17&nb_items=7&group_cancer=1&include_nmsc=1&include_nmsc_other=1&half_pie=0&donut=0. Accessed 30 Nov 2022.

[18] Taghipour Zahir SH, Aalipour E, Barand P (2015). Interrelationships between Ki67, HER2/neu, p53, ER, and PR status and their associations with tumor grade and lymph node involvement in breast carcinoma subtypes: retrospective-observational analytical study. Medicine.

[19] Alipour S, Omranipour R, Jahanzad I, Bagheri K (2013). Very young breast cancer in a referral center in Tehran, Iran: Review of 55 Cases Aged 25 or Less throughout 33 Years. Asian Pac J Cancer Prev.

[20] Tran A, Pio BS, Khatibi B, Czernin J, Phelps ME, Silverman DH (2005). 18F-FDG PET for staging breast cancer in patients with inner-quadrant versus outer-quadrant tumors: comparison with long-term clinical outcome. J Nucl Med.

[21] Perkins CI, Hotes J, Kohler BA, Howe HL (2004). Association between breast cancer laterality and tumor location, United States, 1994–1998. Cancer Causes Control.

[22] Mighri N, Mejri N, Boujemaa M, Berrazega Y, Rachdi H, El Benna H (2022). Association between epidemiological and clinicopathological features of breast cancer with prognosis, family history, Ki-67 proliferation index and survival in Tunisian breast cancer patients. PLoS One.

[23] Wu S, Zhou J, Ren Y, Sun J, Li F, Lin Q (2014). Tumor location is a prognostic factor for survival of Chinese women with T1–2N0M0 breast cancer. Int J Surg.

[24] Sohn VY, Arthurs ZM, Sebesta JA, Brown TA (2008). Primary tumor location impacts breast cancer survival. Am J Surg.

[25] Kroman N, Wohlfahrt J, Mouridsen HT, Melbye M (2003). Influence of tumor location on breast cancer prognosis. Int J Cancer.

[26] Al Tamimi DM, Shawarby MA, Ahmed A, Hassan AK, AlOdaini AA (2010). Protein expression profile and prevalence pattern of the molecular classes of breast cancer-a Saudi population-based study. BMC Cancer.

[27] Caldarella A, Buzzoni C, Crocetti E, Bianchi S, Vezzosi V, Apicella P (2013). Invasive breast cancer: a significant correlation between histological types and molecular subgroups. J Cancer Res Clin Oncol.

[28] Cherbal F, Gaceb H, Mehemmai C, Saiah I, Bakour R, Rouis AO (2015). Distribution of molecular breast cancer subtypes among Algerian women and correlation with clinical and tumor characteristics: a population-based study. Breast Dis.

[29] Salhia B, Tapia C, Ishak EA, Gaber S, Berghuis B, Hussain KH (2011). Molecular subtype analysis determines the association of advanced breast cancer in Egypt with favorable biology. BMC Women’s Health.

[30] Shibuta K, Ueo H, Furusawa H, Komaki K, Rai Y, Sagara Y (2011). The relevance of intrinsic subtype to clinicopathological features and prognosis in 4,266 Japanese women with breast cancer. Breast Cancer.

[31] Anwar SL, Avanti WS, Dwianingsih EK, Cahyono R, Suwardjo S (2022). Risk Factors, Patterns, and Distribution of Bone Metastases and Skeletal-Related Events in High-Risk Breast Cancer Patients. Asian Pac J Cancer Prev.

[32] Wang R, Zhu Y, Liu X, Liao X, He J, Niu L (2019). The Clinicopathological features and survival outcomes of patients with different metastatic sites in stage IV breast cancer. BMC Cancer.

[33] Al-Gaithy ZK, Yaghmoor BE, Koumu MI, Alshehri KA, Saqah AA, Alshehri HZ (2019). Trends of mastectomy and breast-conserving surgery and related factors in female breast cancer patients treated at King Abdulaziz University Hospital, Jeddah, Saudi Arabia, 2009–2017: A retrospective cohort study. Ann Med Surg (Lond).

[34] Kummerow KL, Du L, Penson DF, Shyr Y, Hooks MA (2015). Nationwide trends in mastectomy for early-stage breast cancer. JAMA Surg.

[35] Dragun AE, Huang B, Tucker TC, Spanos WJ (2012). Increasing mastectomy rates among all age groups for early stage breast cancer: a 10-year study of surgical choice. Breast J.

[36] Najafi M, Ebrahimi M, Kaviani A, Hashemi E, Montazeri A (2005). Breast conserving surgery versus mastectomy: cancer practice by general surgeons in Iran. BMC Cancer.

[37] Han W, Kang SY, Korean Breast Cancer Society (2010). Relationship between age at diagnosis and outcome of premenopausal breast cancer: age less than 35 years is a reasonable cut-off for defining young age-onset breast cancer. Breast Cancer Res Treat..

[38] Hwang KT, Kim J, Jung J, Chang JH, Chai YJ, Oh SW (2019). Impact of breast cancer subtypes on prognosis of women with operable invasive breast cancer: a population-based study using SEER Database Breast cancer subtype and prognosis. Clin Cancer Res.

[39] Nixon AJ, Neuberg D, Hayes DF, Gelman R, Connolly JL, Schnitt S (1994). Relationship of patient age to pathologic features of the tumor and prognosis for patients with stage I or II breast cancer. J Clin Oncol.

[40] Anastasiadi Z, Lianos GD, Ignatiadou E, Harissis HV, Mitsis M (2017). Breast cancer in young women: an overview. Updates Surg.

